# Inferred functional microbiome features are associated with clinical trajectories of low-grade cervical lesions

**DOI:** 10.1186/s12866-026-05648-7

**Published:** 2026-09-12

**Authors:** Milan Stosic, Mariano A. Molina, Dhananjay Mukhedkar, Sadaf Sakina Hassan, Anna Sahlin, Cristina Ruiz Ballester, Jiangrong Wang, Laila Sara Arroyo Mühr

**Affiliations:** 1 Norwegian HPV Reference Laboratory, Department of Microbiology and Infection Control, Akershus University Hospital, Lørenskog, 1478 Norway; 2 Department of Laboratory Medicine, Karolinska Institutet, Huddinge, 141 52 Sweden; 3 Department of Cellular Therapy and Allogeneic Stem Cell Transplantation (CAST), Karolinska University Hospital, Huddinge, Sweden; 4 Center for Cervical Cancer Elimination, F56, Karolinska University Hospital Huddinge, Karolinska Institutet, Huddinge, 141 86 Sweden

**Keywords:** Cervicovaginal microbiome, Human papillomavirus, Low-grade squamous intraepithelial lesions, Community state types, Microbial function, 16S rRNA sequencing, Clinical outcome

## Abstract

**Background:**

The cervicovaginal microbiome has been associated with human papillomavirus (HPV)-related cervical disease, but its role in determining the clinical trajectory of low-grade squamous intraepithelial lesions (LSIL) remains unclear. We investigated whether microbial taxonomic, ecological, and inferred functional features could distinguish LSIL regression from progression and improve risk stratification.

**Results:**

The cervicovaginal microbiome of 90 women with LSIL and known clinical outcomes was profiled using 16S rRNA gene sequencing. Overall microbiome diversity and composition did not differ significantly between regression and progression groups. Instead, microbial profiles clustered primarily by community state type (CST), with CST I-B nominally more frequent among regression cases. Within *Lactobacillus*-dominated communities, CST I-B exhibited distinct inferred functional profiles characterized by enrichment of carbohydrate metabolism and fermentation pathways and relative depletion of nucleotide biosynthesis pathways compared with CST I-A. Exploratory logistic regression models based on age, or microbiome taxa alone, showed limited discriminatory performance, whereas an integrated model incorporating age, microbiome, and inferred functional pathway features improved apparent discrimination between regression and progression in this cohort (AUC = 0.76, 95% CI 0.66–0.86).

**Conclusions:**

Differences in microbial community organization and inferred functional profiles were observed to be associated with LSIL clinical trajectories, whereas taxonomic composition alone showed limited discriminatory value. Integrating microbial taxonomic, ecological, and functional features modestly improved the apparent discrimination of LSIL outcomes, suggesting that inferred functional features may provide complementary information that warrants future research.

**Supplementary Information:**

The online version contains supplementary material available at https://doi.org/10.1186/s12866-026-05648-7.

## Introduction

Cervical cancer remains a major global health burden despite being largely preventable through human papillomavirus (HPV) vaccination and screening programs [1, 2]. Persistent infection with high-risk HPV types is the central driver of cervical carcinogenesis, and HPV-based screening has substantially improved the detection of cervical precancer lesions [3]. However, while HPV infection is highly prevalent, only a minority of infections progress to clinically significant disease, and most HPV infections and low-grade squamous intraepithelial lesions (LSIL) regress spontaneously without intervention [4, 5]. Consequently, current screening strategies identify many women who are unlikely to progress, resulting in repeated follow-up procedures, patient anxiety, increased healthcare costs, and potential overtreatment [6].

A major challenge in cervical cancer prevention is the identification of biomarkers capable of distinguishing lesions with divergent clinical trajectories at an early stage. While cytology and HPV genotyping provide important clinical information, they remain limited in predicting which LSIL cases will regress, and which will persist or progress to higher-grade lesions [6, 7]. Improved risk stratification strategies are therefore needed to better guide clinical management and reduce unnecessary interventions.

The cervicovaginal microbiome has emerged as a potential contributor to HPV persistence and cervical disease progression [8, 9]. The cervicovaginal microbiota is commonly structured into community state types (CSTs), in which specific bacterial species dominate the microbial ecosystem or assemble into polymicrobial anaerobic communities [10, 11]. *Lactobacillus*-dominated CSTs are generally associated with cervicovaginal homeostasis, whereas *Lactobacillus*-depleted, polymicrobial communities enriched with anaerobic bacteria have been linked to bacterial vaginosis, persistent HPV infection, and cervical neoplasia [8, 12, 13]. In particular, polymicrobial communities have been frequently associated with viral persistence, disease progression, and high-grade cervical lesions [14, 15].

Increasing evidence suggests that *Lactobacillus*-dominated communities are not biologically equivalent. Subgroups within CSTs may exhibit distinct ecological and metabolic characteristics regardless of sharing dominant taxa [11, 16]. For example, CST I subtypes, such as CST I-A and CST I-B, differ in the abundance of non-dominant bacterial species and may represent functionally distinct microbial environments [11, 16, 17]. These observations suggest that microbial functional activity may contribute to divergent HPV-related clinical outcomes [18, 19]. Most microbiome studies in cervical disease, however, have been cross-sectional and focused primarily on taxonomic differences between disease stages. Thus, it remains unclear whether baseline taxonomic and inferred functional features, particularly within CST I subtypes, are associated with the subsequent regression or progression of LSIL. Clinical outcomes determined through longitudinal follow-up are therefore essential for evaluating whether baseline microbiome features are associated with subsequent lesion trajectories.

Recent advances in microbiome research and computational biology have enabled the integration of microbial taxonomic, ecological, and functional features to provide a more comprehensive representation of cervicovaginal microbial communities [20, 21]. Predictive modelling approaches can be used to evaluate whether combinations of microbial characteristics provide additional information about clinical outcomes beyond individual features alone. In cervical disease, models incorporating microbiome-derived features have shown promise for improving risk stratification and identifying microbial signatures associated with HPV persistence and lesion progression [14, 22, 23]. However, the potential value of combining taxonomic, ecological, and functional microbiome features to distinguish regression from progression in women with LSIL remains insufficiently explored.

In this study, we characterized the cervicovaginal microbiome in women with LSIL and known clinical outcomes using 16S rRNA gene sequencing and inferred functional profiling. We investigated microbial diversity, taxonomic composition, CSTs, and inferred microbial functional pathways associated with lesion regression and progression. We further evaluated whether microbiome-derived taxonomic and inferred functional features could discriminate clinical trajectories using logistic regression models. Our analysis identified exploratory differences in inferred functional profiles within *Lactobacillus*-dominated communities and evaluated their potential contribution to discrimination between LSIL outcomes.

## Methods

### Study design, clinical data, and sample selection

A retrospective case–control study was conducted within the Stockholm Cervical Screening Programme using cervical samples identified through the Swedish National Cervical Screening Registry (NKCx, nkcx.se) and subsequently retrieved from the Stockholm Cervical Screening Biobank between 2013 and 2023 [24]. Cases were linked to biobank samples using personal identification numbers. Screening samples were identified based on a cytological diagnosis of LSIL. For women aged 23–27 years, the index sample corresponded to the screening sample with LSIL cytology. For women aged 28–65 years, the index sample was defined as the screening sample collected within six months before a histologically confirmed LSIL diagnosis. This approach reflects clinical management guidelines in place at the time, whereby younger women with LSIL cytology were not routinely referred for colposcopy and histopathological assessment.

Clinical outcomes were obtained from NKCx follow-up data, including both cytology and histology records available through the end of 2023, and no fixed follow-up window was applied. Progression was defined as the development of HSIL+, including CIN2/3, adenocarcinoma in situ, or invasive cervical cancer, during follow-up. Regression was defined as the absence of any HSIL+ diagnosis after the initial LSIL and a final recorded status of HPV negativity with either normal cytology or no cytological abnormalities by the end of follow-up. A total of 21,759 LSIL samples were identified in the biobank. Among these, 4,619 progressed to HSIL + and 10,062 regressed during follow-up. From this population, 200 samples were initially selected for downstream analyses, stratified by age group (23–50 and 51–65 years) and clinical outcome. For this proof-of-concept study, 90 samples were selected before library preparation by prioritizing samples with higher available DNA concentrations while maintaining equal numbers of regression and progression cases (45 per group). The selected samples had a median DNA concentration of 8.75 ng/µL, compared with 1.97 ng/µL among the remaining samples. Therefore, the remaining 110 samples were not processed in this study. Available HPV test results corresponding to the baseline LSIL sample were retrieved from clinical records and categorized as HPV16/18-positive, positive for other known high-risk HPV types, generic high-risk HPV-positive without genotype-specific information, or high-risk HPV-negative.

### DNA extraction, library preparation, and sequencing

Aliquots of 100 µL were obtained from the Stockholm Cervical Screening Biobank. DNA extraction was performed using a Hamilton Microlab STAR liquid handling robot (Hamilton Company, Reno, NV, USA). DNA concentration was measured using the Qubit™ 1X dsDNA High Sensitivity Assay Kit (Thermo Fisher Scientific) on a Qubit 4 Fluorometer. Samples were stored at − 20 °C between processing steps. Prior to amplification, DNA concentrations were normalized to 3.5 ng/µL.

Microbiome profiling was performed using 16S rRNA gene amplicon sequencing. Library preparation was carried out at the National Genomics Infrastructure (NGI), SciLifeLab (Stockholm, Sweden), using a two-step PCR protocol targeting the V3–V4 hypervariable regions of the 16S rRNA gene. In the first PCR, locus-specific primers (341 F/805R) with 5′ overhang adapter sequences were used. Amplification was performed using KAPA HiFi HotStart ReadyMix (Roche) under the following conditions: initial denaturation at 98 °C for 2 min, followed by 28 cycles of denaturation at 98 °C for 20 s, annealing at 54 °C for 20 s, and extension at 72 °C for 15 s, with a final extension at 72 °C for 2 min.

PCR products were purified using magnetic bead-based methods. A second PCR was performed to add dual indices and Illumina sequencing adapters, followed by an additional purification step. Library quality was assessed based on fragment size distribution and DNA concentration. Samples with concentrations above 1 ng/µL were considered to have passed quality control. Negative controls (no-template controls) and positive controls were included in each sequencing batch to monitor potential contamination and assay performance. Sequencing was then performed on an Illumina NextSeq 2000 platform (Illumina, USA) using paired-end reads (2 × 151 bp) and dual 10 bp index reads on a P1 flow cell, according to the manufacturer’s instructions. All samples included in the study were processed and sequenced together in a single sequencing batch.

### Bioinformatic analysis

Raw sequencing data were processed using the nf-core/ampliseq pipeline (version 2.17.0). Quality filtering, denoising, chimera removal, and amplicon sequence variant (ASV) inference were performed using DADA2. Taxonomic classification was conducted using the SILVA reference database (version 138.2). Output files, including ASV abundance tables, representative sequences, taxonomic assignments, and phylogenetic trees, were used for downstream analyses. Samples with low sequencing depth (< 10,000 reads) were excluded.

### Microbiome data analysis

All downstream analyses were performed in R v4.3.3 using packages including phyloseq, vegan, pheatmap, ggplot2, pROC, and tidyverse. The phylogenetic tree was constructed with the interactive Tree Of Life (iTOL) tool (https://itol.embl.de). Alpha diversity was calculated using the Shannon index. Microbial community structure was explored using principal coordinates analysis (PCoA) based on species-level relative abundance profiles. Taxonomic composition was summarized at genus and species levels, and relative abundance data were visualized using heatmaps with log transformation and scaling. Dominance of microbial species was defined by highest relative abundance. CSTs were classified into I, II, III, IV and V as described by Ravel et al. and France et al. [10, 11], who defined microbial communities based on microbiome composition. We next performed unsupervised clustering analyses and compared the clusters to known CSTs and CST subtypes, as performed previously [16].

### Inferred functional composition

Functional potential of the microbiome was inferred using PICRUSt2 [25] based on ASV profiles. Predicted gene and family abundances were mapped to MetaCyc pathways. Differential pathway abundance analyses were performed between groups and between CST subtypes, and effect sizes were expressed as log2 fold changes. Functional modules were calculated by aggregating log-transformed abundances of biologically related PICRUSt2-inferred MetaCyc pathways into broader metabolic categories, including carbohydrate metabolism and fermentation/SCFA-associated functions.

### Correlation and network analysis

Associations between bacterial taxa and functional pathways were assessed using Spearman correlation analysis. Exploratory taxon association networks were constructed for CST I-A and CST I-B using a shared taxon set derived from the 25 most abundant species and restricted to species detected in at least 10% of samples across the combined CST I-A and CST I-B cohort. Edges were defined by an absolute Spearman correlation coefficient |ρ| ≥ 0.65, using the same threshold in both subtypes. Network connectivity was summarized using edge count, network density, and mean degree.

### Logistic regression analysis

Logistic regression was used to evaluate whether microbiome-derived features could discriminate between regression and progression outcomes. Models incorporated species-level taxa, age, and inferred functional pathway scores, either individually or in combined feature sets. Models were evaluated using age alone, microbiome taxa alone, or an integrated feature set combining age, microbiome taxa, and inferred functional pathway scores. Model performance was evaluated using receiver operating characteristic (ROC) curves and area under the curve (AUC) with 95% confidence intervals. Model performance was assessed in the same cohort used for model development. No internal cross-validation, independent test set, or external validation was performed; therefore, the reported AUCs represent apparent within-cohort discrimination.

### Statistics and reproducibility

All statistical analyses were conducted in R v4.3.3. *p*-values < 0.05 were considered statistically significant. Comparisons between categorical variables were performed using Fisher’s exact test, whereas continuous variables between two groups were compared using Mann–Whitney U tests. For analyses involving multiple taxa, pathways, or correlations, *p*-values were adjusted using the Benjamini–Hochberg false discovery rate (FDR) correction. Differences in connectivity between CST I-A and CST I-B were evaluated using one-sided permutation tests with 9,999 permutations, in which samples were randomly reassigned between groups while preserving the original group sizes. Pairwise comparisons of ROC curves were performed using DeLong’s test. All analyses were based on single measurements per sample, and no technical replicates were included.

## Results

### Study design and cohort characteristics

**Fig. 1:**
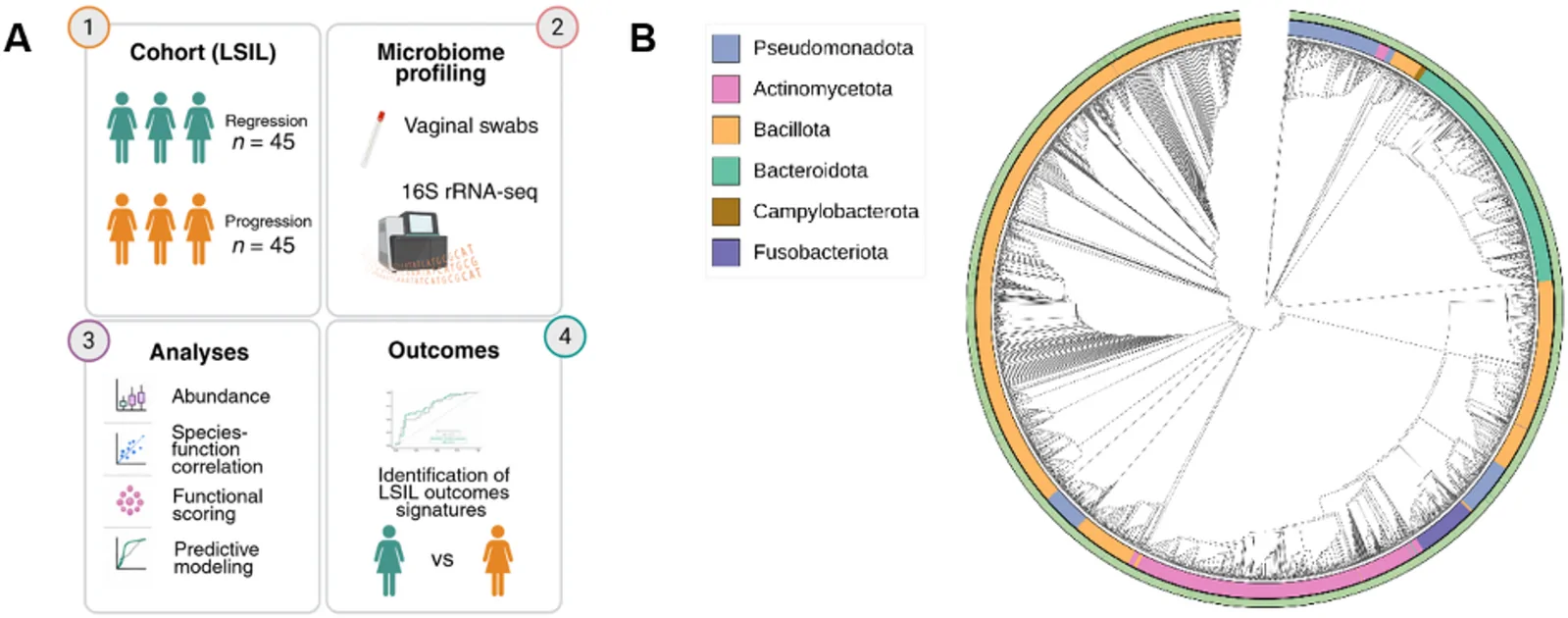
Study overview and microbiome profiling of the LSIL cohort. **A** Women diagnosed with low-grade squamous intraepithelial lesions (LSIL) were followed and stratified based on clinical outcome into regression (n = 45) and progression (n = 45). Cervicovaginal samples were collected and subjected to 16S rRNA gene sequencing (16S rRNA-seq) for microbiome profiling. Three samples were subsequently excluded because of low sequencing depth, leaving 87 samples for downstream analyses. Downstream analyses included differential abundance testing, species-function correlation, inferred functional pathway scoring using PICRUSt2, and regression analysis to assess microbial signatures associated with disease outcome. **B** Phylogenetic composition of the cervicovaginal microbiome across the cohort, visualized as a circular tree with taxa colored by major bacterial phyla

The study cohort comprised 90 women diagnosed with LSIL, of whom 45 regressed and 45 progressed during follow-up (Fig. 1A). Among women who progressed, the median interval from index-sample collection to HSIL+ diagnosis was 0.42 years (IQR, 0.30–1.05; range, 0.15–6.94 years). The overall taxonomic composition of the cervicovaginal microbiota demonstrated the diversity of bacterial taxa across the cohort (Fig. 1B). Sequencing depth did not differ substantially between regression and progression groups (Supplementary Fig. S1A). Three samples had low sequencing depth (<10,000 reads) and were excluded from downstream analyses, resulting in a final analytical cohort of 87 samples, including 44 regression and 43 progression cases. Baseline HPV16/18 positivity did not differ significantly between progression and regression cases (16/45 [35.6%] vs 12/45 [26.7%]; Fisher’s exact test, p = 0.495; Supplementary Table S1). Among participants with genotype-informative high-risk HPV results, the distribution of HPV16/18 versus other high-risk HPV types was also similar between outcomes (p = 0.359). Participant age did not differ significantly between groups (median age 49 years [range 24–65] in the regression group vs 38 years [range 23–63] in the progression group; Mann–Whitney U test, p = 0.074; Supplementary Fig. S1B).

### Microbiome structure and community state type clasifications

**Fig. 2:**
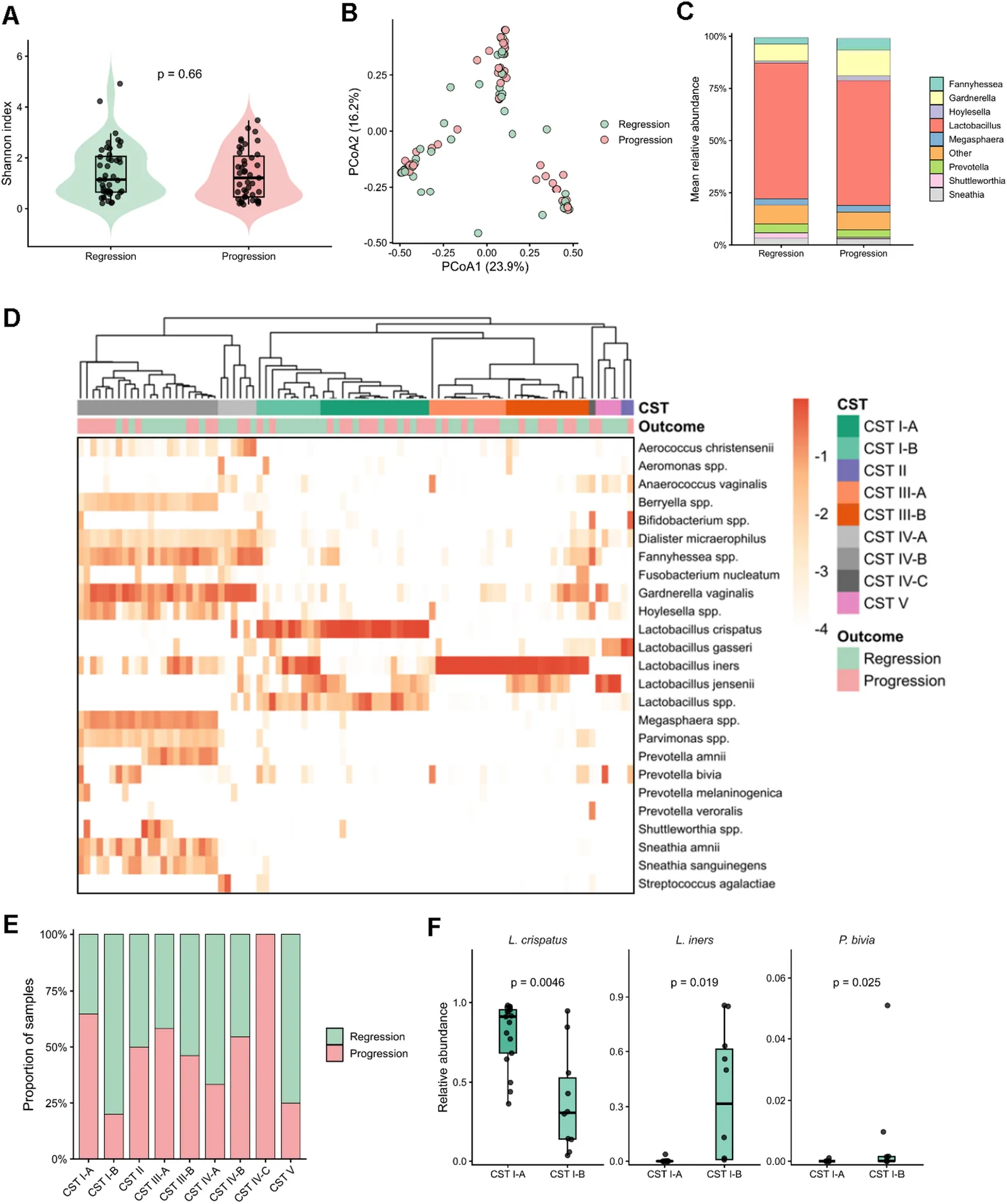
Cervicovaginal microbiome structure and key taxa associated with LSIL outcomes. **A** Alpha diversity (Shannon index) comparing regression and progression groups (Mann–Whitney U test). **B** Principal coordinates analysis (PCoA) based on Bray–Curtis distances of species-level microbiome profiles. **C** Relative abundance of dominant bacterial genera across outcomes. **D** Heatmap of species-level relative abundances (log-transformed and scaled), clustered by samples and taxa. Annotation bars indicate community state types (CST) and clinical outcomes. **E** Distribution of CSTs across regression and progression groups. **F** Relative abundance of key taxa associated with CST I subtypes. *p*-values were adjusted for multiple testing using the Benjamini–Hochberg method

Global microbiome composition was first examined to determine whether it differed between LSIL regression and progression groups. Alpha diversity (Shannon index) did not differ significantly between regression and progression groups (Fig. 2A). Similarly, principal coordinates analysis (PCoA) based on Bray–Curtis distances of species-level profiles revealed substantial overlap between outcomes, with no clear separation between regression and progression samples (Fig. 2B). Although beta dispersion analysis did not reach statistical significance, a small difference in within-group microbiome variability was observed between outcomes (Mann–Whitney U test, *p* = 0.062; Supplementary Fig. S2). Genus-level composition was also similar across groups (Fig. 2C).

Higher-order microbial community organization was investigated to determine whether it could better capture differences associated with clinical outcome. CST classification revealed distinct structural organization within the cohort, with samples clustering primarily according to CST composition (Fig. 2D, Supplementary Table S2). CST distribution was not significantly associated with clinical outcome overall (Fisher’s exact test with Monte Carlo simulation, *p* = 0.386; Supplementary Table S2). Although regression was more frequent in CST I-B than in CST I-A (8/10 [80.0%] versus 6/17 [35.3%]), this exploratory pairwise difference was only nominally significant and did not remain significant after correction for multiple comparisons (Fisher’s exact test, *p* = 0.0467; BH-adjusted *p* = 1.00). The contrasting distributions of these two CST I subtypes motivated further exploratory comparisons. Within CST I, subtype-specific differences were observed in both diversity and composition. CST I-B samples exhibited significantly higher alpha diversity compared to CST I-A (Mann–Whitney U test, *p* = 0.031; Supplementary Fig. S3A), consistent with a more heterogeneous microbial community. In contrast, CST I-A samples displayed higher *Lactobacillus* dominance, although this difference was not significant (Mann–Whitney U test, *p* = 0.052; Supplementary Fig. S3B). To further describe the composition of CST I-A and CST I-B, we also compared the relative abundances of their predominant taxa (Fig. 2F). Species-level analyses showed that *Lactobacillus crispatus* was more abundant in CST I-A, whereas *Lactobacillus iners* and *Prevotella bivia* were enriched in CST I-B (*p* < 0.05, Fig. 2F).

To further characterize the ecological organization of these CST I subtypes, co-occurrence network analyses were performed. CST I-A exhibited a relatively structured and cohesive microbial network with strong co-occurrence patterns (Supplementary Fig. S4). In contrast, CST I-B displayed a greater number of co-occurrence relationships involving a broader range of anaerobic taxa (Supplementary Fig. S5). CST I-B showed 13 edges and a network density of 0.096, compared with 2 edges and a density of 0.015 in CST I-A. However, this apparent increase in network connectivity was not statistically significant in a permutation analysis (*p* = 0.23).

### Inferred functional pathway profiles across community state types

To investigate the functional potential of the microbiome, MetaCyc pathway abundances were inferred using PICRUSt2. Like the taxonomic analyses, functional profiles clustered primarily according to CSTs (Supplementary Fig. S6), revealing that microbial community structure strongly influenced the predicted metabolic landscape.

**Fig. 3:**
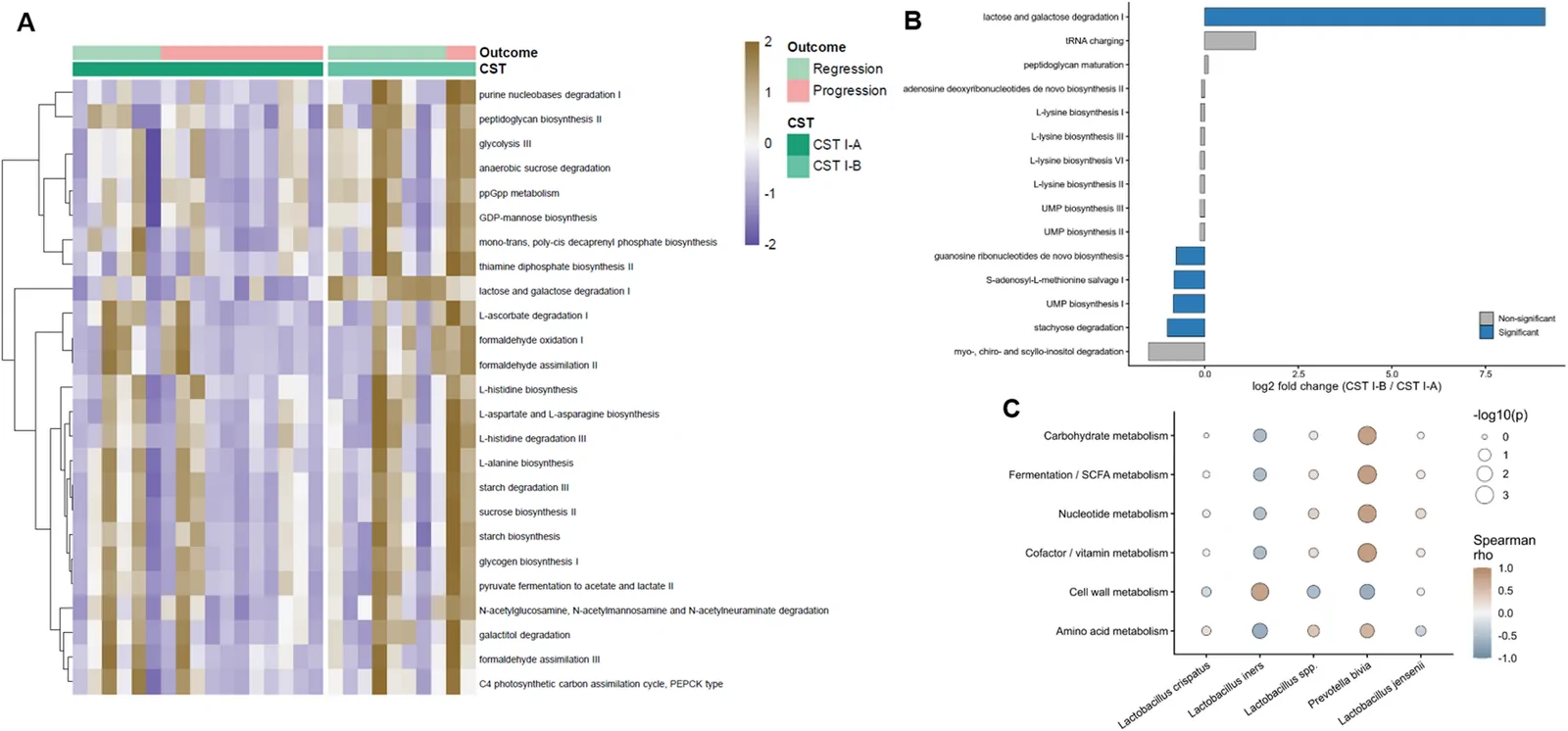
Inferred functional pathway alterations and species-function associations in CST I subtypes. **A** Heatmap of MetaCyc pathway abundances inferred using PICRUSt2 (log-transformed and row-scaled) across CST I samples. Samples are clustered based on inferred functional pathways, with annotation bars indicating clinical outcome (regression vs. progression) and community state type (CST). **B** Top differentially abundant pathways between CST I-B and CST I-A, ranked by effect size (log2 fold change). **C** Associations between selected CST I-B-resident species and functional modules, shown as Spearman correlations. Dot size represents statistical significance (log10 *p*-value), and color indicates correlation strength and direction

Following the nominal enrichment of CST I-B among LSIL regression cases, inferred functional differences within *Lactobacillus*-dominated communities were next examined. Comparative analyses between CST I-B and CST I-A revealed distinct inferred pathway-level differences, with significant enrichment of lactose and galactose degradation I in CST I-B, whereas pathways related to nucleotide biosynthesis were relatively depleted (Fig. 3A and B, Supplementary Table S3). Consistent with these observations, inferred functional module analysis demonstrated increased activity of carbohydrate metabolism and fermentation/short-chain fatty acid (SCFA) pathways in CST I-B compared to CST I-A (Supplementary Fig. S7A). In contrast, inferred pathway differences between regression and progression groups at the cohort level were limited and did not reach statistical significance (Supplementary Fig. S7B), further supporting the notion that functional heterogeneity was more apparent within specific CSTs.

To further explore the relationship between microbial composition and predicted metabolic activity, correlations between selected bacterial species and inferred functional modules were examined within CST I-B samples. Strong positive associations were observed between anaerobic taxa, including *Prevotella bivia*, and pathways related to carbohydrate metabolism and fermentation (Fig. 3C). Likewise, *Lactobacillus iners* displayed a significant positive association with cell wall metabolism (Fig. 3C). In contrast, no statistically significant associations were observed between the unresolved *Lactobacillus* spp. category and the inferred functional pathways examined. *L. crispatus* and *L. jensenii* showed weaker and more variable associations.

### Regression analysis using microbiome and inferred functional features

**Fig. 4:**
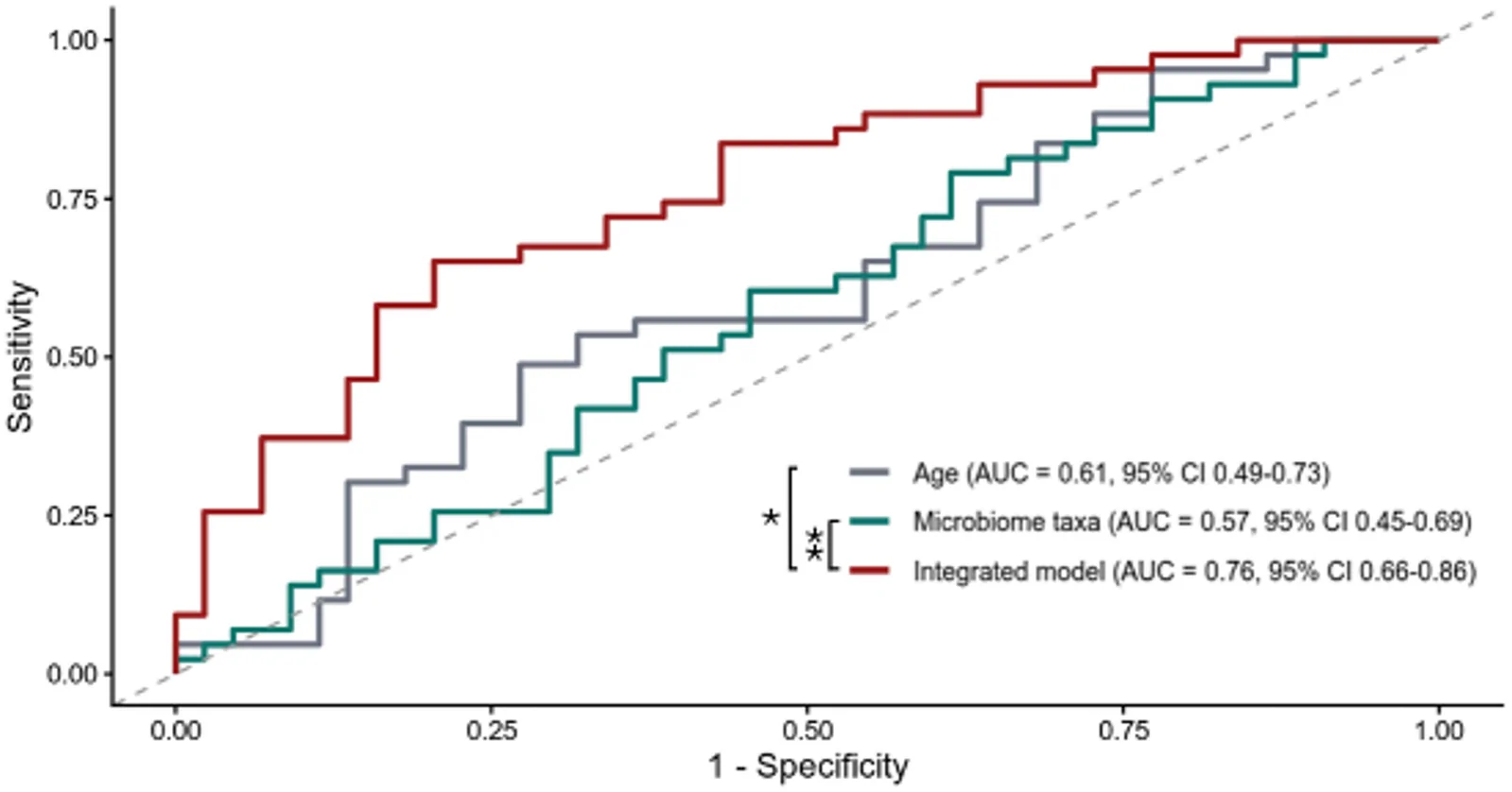
Regression analysis using microbiome and inferred functional features. ROC curves comparing logistic regression models for predicting LSIL clinical outcomes (regression vs. progression) using age alone, microbiome taxa alone, or an integrated model combining age, microbiome taxa, and inferred functional features (*n* = 87). AUC values and 95% confidence intervals are shown for each model. Comparisons of ROC curves were performed using paired DeLong tests within the model-development cohort. * *p* < 0.05; ** *p* < 0.01

Because taxonomic and inferred functional differences were observed within microbial communities, microbiome-derived features were next explored for their ability to discriminate LSIL outcomes using logistic regression models. Models incorporating age or microbiome taxa showed near-random discrimination between regression and progression groups (AUCs = ~ 0.57–0.61; Fig. 4). The integrated model combining age, microbiome taxa, and inferred functional pathway scores showed higher apparent discrimination (AUC = 0.76, 95% CI 0.66–0.86; Fig. 4) compared with the age-only model (DeLong test, *p* = 0.015), and the microbiome taxa model (*p* = 0.006). However, these comparisons were performed within the model-development cohort and do not constitute internal or external validation.

## Discussion

The cervicovaginal microbiome has been increasingly associated with HPV persistence and cervical disease, although its role in determining lesion trajectory remains incompletely understood. In this study, we investigated whether microbiome-derived taxonomic and inferred functional features were associated with LSIL regression or progression. Although overall microbiome diversity and global taxonomic composition did not differ substantially between clinical outcomes, microbial community organization and inferred functional signatures revealed more nuanced patterns associated with lesion behavior.

An important observation of this study was that microbial profiles were structured primarily according to CSTs, with CST I-B nominally more frequent among regression cases. This finding highlights the importance of considering broader microbial community organization in HPV-associated disease. *Lactobacillus*-dominated communities are generally considered markers of cervicovaginal health, whereas *Lactobacillus*-depleted, polymicrobial communities have been linked to HPV persistence and cervical neoplasia [12, 14]. However, increasing evidence suggests that *Lactobacillus*-dominated microbiomes are not biologically homogeneous [11, 16]. Our findings extend this concept by demonstrating that CST I subtypes showed differences in taxonomic composition and inferred metabolic profiles, together with descriptive differences in ecological organization.

Specifically, CST I-B exhibited enrichment of lactose and galactose degradation pathways together with broader shifts involving carbohydrate metabolism and fermentation-related functions, whereas several nucleotide biosynthesis pathways were relatively reduced [26, 27]. These inferred pathways could influence cervicovaginal homeostasis by altering the local production of lactate and other microbial metabolites, which may affect epithelial barrier integrity, inflammation, and antiviral immune responses, including dendritic-cell and T-cell functions relevant to HPV clearance [19, 28–32]. Collectively, these observations suggest that functionally distinct metabolic states may exist within *Lactobacillus*-dominated microbiomes. Such functional variation may influence the cervicovaginal microenvironment through modulation of nutrient availability, epithelial interactions, or microbial cross-feeding networks [18, 28]. Interestingly, integrative metagenomic and metatranscriptomic studies support the concept that vaginal microbial communities exhibiting similar taxonomic composition may display distinct transcriptional and metabolic states [33–35]. These observations are consistent with our findings that inferred CST-associated functional organization may capture biologically relevant features associated with LSIL outcomes. Although causal relationships cannot be inferred from this study, our findings support the view that inferred functional profiles may also be important, alongside taxonomic composition, in understanding host-microbiome interactions associated with cervical disease [33–35].

The observed differences in co-occurrence patterns between CST I subtypes further support this interpretation. Whereas CST I-A exhibited a relatively organized microbial structure dominated by *Lactobacillus* species, the CST I-B network displayed a greater number of co-occurrence relationships involving anaerobic taxa such as *P. bivia* [14]. These observations suggest that subtle shifts in microbial interactions may contribute to distinct metabolic environments within *Lactobacillus*-dominated communities. Likewise, CST I-B was characterized by enrichment of *L. iners* and *P. bivia*, taxa that are often associated with transitional or non-optimal cervicovaginal states [17]. This is consistent with longitudinal studies showing persistent enrichment of dysbiosis-associated anaerobes, including *P. bivia*, *Sneathia*, and *Megasphaera*, during cervical disease and after treatment, supporting a model where cervicovaginal dysbiosis may represent an underlying ecological predisposition associated with cervical disease dynamics [36, 37]. However, despite the presence of these bacteria, CST I-B was nominally enriched among regression cases in our cohort, implying that the biological impact of these taxa may depend on the broader ecological and functional context in which they occur.

Recent evidence suggests that *Prevotella* species contribute substantially to vaginal metabolic activity and sialidase-associated functions across multiple CSTs, including *Lactobacillus*-associated communities, demonstrating that non-dominant anaerobic taxa may disproportionately shape cervicovaginal functional states [38, 39]. Likewise, we found *L. iners* associated with pathways related to cell wall and membrane metabolism, supporting previous observations that *Lactobacillus*-dominated communities can differ substantially in their ecological and functional properties [18, 40]. This observation is consistent with recent metagenomic data demonstrating that strain-level and functional heterogeneity within vaginal microbial communities may influence host interactions and disease-associated states independently of overall CST classification [34]. Taken together, our findings raise the possibility that CST I-B may represent a functionally distinct *Lactobacillus*-associated ecosystem with potential relevance to HPV persistence biology [16, 17, 41].

Consistent with these observations, incorporation of inferred functional pathway information improved apparent within-cohort discrimination for LSIL outcomes. Although overall discrimination remained moderate, these results indicate that inferred functional microbiome profiling may provide complementary information for further evaluation, as similarly observed by other studies [42, 43]. This moderate discrimination also suggests that microbiome-derived features capture only part of the biological variation underlying LSIL trajectories, which are likely influenced by additional host and microenvironmental factors, including immune, transcriptomic, and epigenetic processes [44, 45]. More broadly, our findings support the potential utility of integrative modelling frameworks for combining multidimensional microbiome features that may not be captured through conventional single-feature analyses. These results, however, do not imply that CSTs represent discrete biological entities but instead suggest that microbiome-associated risk may vary according to the broader ecological and functional organization of the cervicovaginal microbiome [46]. From a translational perspective, microbiome-based prediction models may therefore benefit from incorporating ecological context and inferred functional states across highly heterogeneous and dynamic cervicovaginal microbial communities [14, 46–48].

Our study has several limitations that should be considered. The relatively small cohort size may have limited statistical power, influencing the robustness of the modelling analyses. In addition, model performance was evaluated in the same cohort used for model development, without internal cross-validation or independent external validation. The reported AUCs may therefore be optimistic and should be interpreted as apparent within-cohort discrimination. Validation in larger independent cohorts is required before the predictive performance of these models can be established. Furthermore, the study focused on clearly defined regression and progression outcomes and excluded persistent LSIL or other intermediate trajectories. This selection of clinically distinct groups may have increased the apparent separation between outcomes and resulted in more favorable estimates of model discrimination. Moreover, the small sample sizes within individual CSTs, particularly CST I-B, reduce statistical power and may yield unstable estimates. Because CST I-B was selected for further characterization after contrasting outcome distributions were observed, the subsequent analyses should be considered exploratory. In addition, functional profiling was inferred using PICRUSt2 and therefore reflects predicted metabolic potential instead of directly measured microbial activity, which will require confirmation using metagenomic, metatranscriptomic, or metabolomic approaches [30]. Of note, Carter et al. have reported poor agreement between PICRUSt2 predictions and shotgun metagenomic profiles in *L. iners*-dominated vaginal communities, which is particularly relevant because *L. iners* was enriched in CST I-B in our study [49]. Likewise, the cross-sectional and single baseline nature of microbiome sampling also prevented assessment of temporal microbial dynamics that may influence lesion regression or progression over time, thereby limiting its reliability for exploring the association with lesion trajectories. Similarly, characteristic taxa of CSTs, such as BVAB1, were not resolved by the taxonomic classification pipeline, which may have limited the characterization of microbial communities [11]. Finally, complete genotype-specific HPV information and other potentially relevant confounding variables, including hormonal status, menstrual cycle, and antibiotic exposure, were not available for analysis [50]. Despite these limitations, the study benefits from well-characterized clinical trajectories derived from a longitudinal screening cohort and from the integration of taxonomic, ecological, and inferred functional microbiome analyses. Together, these approaches provide a more comprehensive view of cervicovaginal microbial organization in relation to LSIL outcome.

## Conclusions

In conclusion, overall microbiome composition alone did not distinguish LSIL regression from progression, whereas microbial community structure and inferred functional profiles revealed distinct patterns, particularly within *Lactobacillus*-dominated CSTs. These findings highlight the importance of considering microbial functional states and ecological context in studies of cervical disease and support the potential value of inferred functional microbiome features for future research in microbiome-based risk stratification strategies.

## Supplementary Information

Supplementary Material 1.

## Data Availability

Sequencing data have been deposited in the NCBI Sequence Read Archive (SRA) under BioProject accession PRJNA1476274. Custom R scripts used for data processing, statistical analyses, and figure generation are available from the corresponding author upon reasonable request.

## Abbreviations

ASV: Amplicon sequence variant
AUC: Area under the curve
CI: Confidence interval
CST: Community state type
FDR: False discovery rate
HPV: Human papillomavirus
HSIL: High-grade squamous intraepithelial lesion
LSIL: Low-grade squamous intraepithelial lesion
MetaCyc: Metabolic Pathway Database
NKCx: Swedish National Cervical Screening Registry
PCoA: Principal coordinates analysis
PICRUSt2: Phylogenetic Investigation of Communities by Reconstruction of Unobserved States 2
ROC: Receiver operating characteristic

## References

[1] Singh D, Vignat J, Lorenzoni V, Eslahi M, Ginsburg O, Lauby-Secretan B, et al. Global estimates of incidence and mortality of cervical cancer in 2020: a baseline analysis of the WHO Global Cervical Cancer Elimination Initiative. Lancet Global Health. 2023;11(2):e197–206. 36528031 10.1016/S2214-109X(22)00501-0 PMC9848409

[2] Wei F, Georges D, Man I, Baussano I, Clifford GM. Causal attribution of human papillomavirus genotypes to invasive cervical cancer worldwide: a systematic analysis of the global literature. Lancet. 2024;404(10451):435–44. 39097395 10.1016/S0140-6736(24)01097-3

[3] Arroyo Mühr LS, Gini A, Yilmaz E, Hassan SS, Lagheden C, Hultin E, et al. Concomitant human papillomavirus (HPV) vaccination and screening for elimination of HPV and cervical cancer. Nat Commun. 2024;15(1):3679. 38693149 10.1038/s41467-024-47909-x PMC11063066

[4] the Ipc, Lycke KD, Steben M, Garland SM, Woo YL, Cruickshank ME, et al. An updated understanding of the natural history of cervical human papillomavirus infection—clinical implications. American Journal of Obstetrics & Gynecology. 2025;232(5):453 – 60. 10.1016/j.ajog.2025.02.029 39983886

[5] He S, Zhu G, Zhou Y, Yang B, Wang J, Wang Z, et al. Predictive models for personalized precision medical intervention in spontaneous regression stages of cervical precancerous lesions. J Translational Med. 2024;22(1):686. 10.1186/s12967-024-05417-y PMC11282852 39061062

[6] Sroczynski G, Esteban E, Widschwendter A, Oberaigner W, Borena W, von Laer D, et al. Reducing overtreatment associated with overdiagnosis in cervical cancer screening—A model-based benefit–harm analysis for Austria. Int J Cancer. 2020;147(4):1131–42. 31872420 10.1002/ijc.32849

[7] Kang WD, Ju UC, Kim SM. Human papillomavirus genotyping for predicting disease progression in women with biopsy-negative or cervical intraepithelial neoplasia grade 1 of low-grade intraepithelial lesion cytology. Int J Gynecol Cancer. 2024;34(1):12–8. 38123190 10.1136/ijgc-2023-004902

[8] Mitra A, Gultekin M, Burney Ellis L, Bizzarri N, Bowden S, Taumberger N, et al. Genital tract microbiota composition profiles and use of prebiotics and probiotics in gynaecological cancer prevention: review of the current evidence, the European Society of Gynaecological Oncology prevention committee statement. Lancet Microbe. 2024;5(3):e291–300. 38141634 10.1016/S2666-5247(23)00257-4

[9] Chen M, Qi C, Qing W, Zhou Z, Zhang Y, Chen R, et al. Vaginal microbiome and sexually-transmitted pathogens in Chinese reproductive-age women: a multicentre cross-sectional and longitudinal cohort study. Nat Commun. 2025;16(1):10002. 41238551 10.1038/s41467-025-64917-7 PMC12618616

[10] Ravel J, Gajer P, Abdo Z, Schneider GM, Koenig SSK, McCulle SL, et al. Vaginal microbiome of reproductive-age women. Proc Natl Acad Sci. 2011;108(supplement1):4680–7. 20534435 10.1073/pnas.1002611107 PMC3063603

[11] France MT, Ma B, Gajer P, Brown S, Humphrys MS, Holm JB, et al. VALENCIA: a nearest centroid classification method for vaginal microbial communities based on composition. Microbiome. 2020;8(1):166. 33228810 10.1186/s40168-020-00934-6 PMC7684964

[12] Osei Sekyere J, Trama J, Adelson M, Trikannad C, DiBlasi D, Schuster R, et al. Lactobacillus-rich cervicovaginal microbiome associated with lower BV, HPV, and cytology outcomes in women. iScience. 2025;28(10):113473. 41019376 10.1016/j.isci.2025.113473 PMC12466286

[13] Hidjo M, Mukhedkar D, Masimirembwa C, Lei J, Arroyo Mühr LS. Cervical cancer microbiome analysis: comparing HPV 16 and 18 with other HPV types. Sci Rep. 2024;14(1):22014. 39317706 10.1038/s41598-024-73317-8 PMC11422507

[14] Molina MA, Leenders WPJ, Huynen MA, Melchers WJG, Andralojc KM. Temporal composition of the cervicovaginal microbiome associates with hrHPV infection outcomes in a longitudinal study. BMC Infect Dis. 2024;24(1):552. 38831406 10.1186/s12879-024-09455-1 PMC11145797

[15] Usyk M, Carlson L, Schlecht NF, Sollecito CC, Grassi E, Wiek F, et al. Cervicovaginal microbiome and natural history of *Chlamydia trachomatis* in adolescents and young women. Cell. 2025;188(4):1051–e6112. 39818212 10.1016/j.cell.2024.12.011 PMC12035847

[16] Molina MA, Andralojc KM, Huynen MA, Leenders WPJ, Melchers WJG. In-depth insights into cervicovaginal microbial communities and hrHPV infections using high-resolution microbiome profiling. npj Biofilms Microbiomes. 2022;8(1):75. 36171433 10.1038/s41522-022-00336-6 PMC9519886

[17] Molina MA, Melchers WJG, Andralojc KM, Leenders WPJ, Huynen MA. Longitudinal analysis on the ecological dynamics of the cervicovaginal microbiome in hrHPV infection. Comput Struct Biotechnol J. 2023;21:4424–31. 37731597 10.1016/j.csbj.2023.09.011 PMC10507478

[18] Lebeau A, Bruyere D, Roncarati P, Peixoto P, Hervouet E, Cobraiville G, et al. HPV infection alters vaginal microbiome through down-regulating host mucosal innate peptides used by Lactobacilli as amino acid sources. Nat Commun. 2022;13(1):1076. 35228537 10.1038/s41467-022-28724-8 PMC8885657

[19] Dai W, Jiang X, Liu Y, Yu Y, Hou J, Xia J et al. Integrated cross-sectional study and functional validation indicate the association of lactobacillus crispatus-derived D-lactic acid with cervical gene expression and precancerous cervical lesions. J Translational Med. 2026. 10.1186/s12967-026-07982-w PMC13085617 41803943

[20] Hernández Medina R, Kutuzova S, Nielsen KN, Johansen J, Hansen LH, Nielsen M, et al. Machine learning and deep learning applications in microbiome research. ISME Commun. 2022;2(1):98. 37938690 10.1038/s43705-022-00182-9 PMC9723725

[21] Gudnadottir U, Prast-Nielsen S, Wagner N, Hugerth LW, Alderheim VK, Antony AT, et al. Machine learning and the role of the vaginal and fecal microbiome in miscarriage: a matched case-control study. npj Biofilms Microbiomes. 2026;12(1):66. 41826313 10.1038/s41522-026-00956-2 PMC13009249

[22] Ilhan ZE, Łaniewski P, Thomas N, Roe DJ, Chase DM, Herbst-Kralovetz MM. Deciphering the complex interplay between microbiota, HPV, inflammation and cancer through cervicovaginal metabolic profiling. eBioMedicine. 2019;44:675–90. 31027917 10.1016/j.ebiom.2019.04.028 PMC6604110

[23] Hu M, Yang W, Yan R, Chi J, Xia Q, Yang Y, et al. Co-evolution of vaginal microbiome and cervical cancer. J Translational Med. 2024;22(1):559. 10.1186/s12967-024-05265-w PMC11167889 38863033

[24] Dillner J, Mühr LSA, Kleppe SN, Wang J, Andersson H, Elfström M, et al. The Swedish Cervical Screening Cohort. Sci Data. 2024;11(1):697. 38926412 10.1038/s41597-024-03519-2 PMC11208431

[25] Douglas GM, Maffei VJ, Zaneveld JR, Yurgel SN, Brown JR, Taylor CM, et al. PICRUSt2 for prediction of metagenome functions. Nat Biotechnol. 2020;38(6):685–8. 32483366 10.1038/s41587-020-0548-6 PMC7365738

[26] Mirzaei R, Kavyani B, Nabizadeh E, Kadkhoda H, Asghari Ozma M, Abdi M. Microbiota metabolites in the female reproductive system: Focused on the short-chain fatty acids. Heliyon. 2023;9(3):e14562. 36967966 10.1016/j.heliyon.2023.e14562 PMC10031489

[27] Mejía-Caballero A, Marco ML. Lactobacilli biology, applications and host interactions. Nat Rev Microbiol. 2026;24(2):111–26. 40702328 10.1038/s41579-025-01205-7

[28] Myeong J, Lee M, Lee B, Kim JH, Nam Y, Choi Y, et al. Microbial metabolites control self-renewal and precancerous progression of human cervical stem cells. Nat Commun. 2025;16(1):2327. 40057497 10.1038/s41467-025-57323-6 PMC11890575

[29] Schwecht I, Nazli A, Gill B, Kaushic C. Lactic acid enhances vaginal epithelial barrier integrity and ameliorates inflammatory effects of dysbiotic short chain fatty acids and HIV-1. Sci Rep. 2023;13(1):20065. 37973920 10.1038/s41598-023-47172-y PMC10654711

[30] Molina MA, Dai W. Microbiome-metabolome crosstalk in HPV pathogenesis: from ecosystem dynamics to translational biomarkers. Computational and Structural Biotechnology Journal. 2026;35:0158. 10.34133/csbj.0158 PMC13329037 42404061

[31] Decout A, Krasias I, Roberts L, Gimeno Molina B, Charenton C, Brown Romero D, et al. *Lactobacillus crispatus *S-layer proteins modulate innate immune response and inflammation in the lower female reproductive tract. Nat Commun. 2024;15(1):10879. 39737998 10.1038/s41467-024-55233-7 PMC11685708

[32] Norenhag J, Edfeldt G, Stålberg K, Garcia F, Hugerth LW, Engstrand L, et al. Compositional and functional differences of the vaginal microbiota of women with and without cervical dysplasia. Sci Rep. 2024;14(1):11183. 38755259 10.1038/s41598-024-61942-2 PMC11099171

[33] France MT, Fu L, Rutt L, Yang H, Humphrys MS, Narina S, et al. Insight into the ecology of vaginal bacteria through integrative analyses of metagenomic and metatranscriptomic data. Genome Biol. 2022;23(1):66. 35232471 10.1186/s13059-022-02635-9 PMC8886902

[34] Holm JB, France MT, Gajer P, Ma B, Brotman RM, Shardell M, et al. Integrating compositional and functional content to describe vaginal microbiomes in health and disease. Microbiome. 2023;11(1):259. 38031142 10.1186/s40168-023-01692-x PMC10688475

[35] Dos Santos SJ, Copeland C, Macklaim JM, Reid G, Gloor GB. Vaginal metatranscriptome meta-analysis reveals functional BV subgroups and novel colonisation strategies. Microbiome. 2024;12(1):271. 39709449 10.1186/s40168-024-01992-w PMC11662499

[36] Banila C, Ladoukakis E, Scibior-Bentkowska D, Santiago LR, Reuter C, Kleeman M, et al. A longitudinal pilot study in pre-menopausal women links cervicovaginal microbiome to CIN3 progression and recovery. Commun Biology. 2025;8(1):883. 10.1038/s42003-025-08328-w PMC12144234 40481153

[37] Mitra A, MacIntyre DA, Paraskevaidi M, Moscicki A-B, Mahajan V, Smith A, et al. The vaginal microbiota and innate immunity after local excisional treatment for cervical intraepithelial neoplasia. Genome Med. 2021;13(1):176. 34736529 10.1186/s13073-021-00977-w PMC8567681

[38] Pelayo P, Hussain FA, Werlang CA, Wu CM, Woolston BM, Xiang CM, et al. Prevotella are major contributors of sialidases in the human vaginal microbiome. Proc Natl Acad Sci. 2024;121(36):e2400341121. 39186657 10.1073/pnas.2400341121 PMC11388281

[39] Segui-Perez C, de Jongh R, Jonkergouw Robin LW, Pelayo P, Balskus Emily P, Zomer A, et al. *Prevotella timonensis* degrades the vaginal epithelial glycocalyx through high fucosidase and sialidase activities. mBio. 2024;15(9):e00691–24. 39162399 10.1128/mbio.00691-24 PMC11389373

[40] Colbert LE, El Alam MB, Wang R, Karpinets T, Lo D, Lynn EJ, et al. Tumor-resident *Lactobacillus iners *confer chemoradiation resistance through lactate-induced metabolic rewiring. Cancer Cell. 2023;41(11):1945–e6211. 37863066 10.1016/j.ccell.2023.09.012 PMC10841640

[41] Molina MA, Melchers WJG, Núñez-Samudio V, Landires I. The emerging role of *Lactobacillus acidophilus* in the cervicovaginal microenvironment. Lancet Microbe. 2024;5(1):e6–7. 37863085 10.1016/S2666-5247(23)00315-4

[42] Tango CN, Seo S-S, Kwon M, Lee D-O, Chang HK, Kim MK. Taxonomic and Functional Differences in Cervical Microbiome Associated with Cervical Cancer Development. Sci Rep. 2020;10(1):9720. 32546712 10.1038/s41598-020-66607-4 PMC7297964

[43] Wu S, Ding X, Kong Y, Acharya S, Wu H, Huang C, et al. The feature of cervical microbiota associated with the progression of cervical cancer among reproductive females. Gynecol Oncol. 2021;163(2):348–57. 34503848 10.1016/j.ygyno.2021.08.016

[44] Herzog C, Vavourakis CD, Barrett JE, Karbon G, Villunger A, Wang J, et al. HPV-induced host epigenetic reprogramming is lost upon progression to high-grade cervical intraepithelial neoplasia. Int J Cancer. 2023;152(11):2321–30. 36810770 10.1002/ijc.34477

[45] Cho M-G, Chang Y, Ryu S. Systemic Immune-Inflammation Markers Associate With Cervical Human Papillomavirus Infection in Asymptomatic Women. Open Forum Infect Dis. 2026;13(4):ofag194. 41983148 10.1093/ofid/ofag194 PMC13075954

[46] Molina MA. From dysbiosis to mechanisms: Why cervicovaginal microbiome-HPV studies must catch up with biology. PLoS Pathog. 2026;21(12):e1013830. 10.1371/journal.ppat.1013830 PMC12752938 41468410

[47] Usyk M, Zolnik CP, Castle PE, Porras C, Herrero R, Gradissimo A, et al. Cervicovaginal microbiome and natural history of HPV in a longitudinal study. PLoS Patho. 2020;16(3):e1008376. 10.1371/journal.ppat.1008376 PMC7098574 32214382

[48] Condori-Catachura S, Ahannach S, Ticlla M, Kenfack J, Livo E, Anukam KC, et al. Diversity in women and their vaginal microbiota. Trends Microbiol. 2025;33(11):1163–72. 39919958 10.1016/j.tim.2024.12.012 PMC12628047

[49] Carter Kayla A, Fodor Anthony A, Balkus Jennifer E, Zhang A, Serrano Myrna G, Buck Gregory A, et al. Vaginal Microbiome Metagenome Inference Accuracy: Differential Measurement Error according to Community Composition. mSystems. 2023;8(2):e01003–22. 36975801 10.1128/msystems.01003-22 PMC10134888

[50] Hugerth LW, Krog MC, Vomstein K, Du J, Bashir Z, Kaldhusdal V, et al. Defining Vaginal Community Dynamics: daily microbiome transitions, the role of menstruation, bacteriophages, and bacterial genes. Microbiome. 2024;12(1):153. 39160615 10.1186/s40168-024-01870-5 PMC11331738

