# Supplementary material for: Inferred functional microbiome features are associated with clinical trajectories of low-grade cervical lesions
Source: BMC Microbiol. 2026 Sep 12;26:802. doi: 10.1186/s12866-026-05648-7 (PMC13570522; doi:10.1186/s12866-026-05648-7)
Supplement: Supplementary file 1 [file 12866_2026_5648_MOESM1_ESM.docx]

**Supplementary Figures**

**Supplementary Figure S1**. **Sequencing depth and age distribution across clinical outcomes**. (**A**) Total sequencing depth per sample, shown as read counts on a log scale for regression and progression groups. (**B**) Age distribution of participants stratified by clinical outcome (regression vs progression). Statistical significance was assessed using a Mann–Whitney U test.

**Supplementary Figure S2. Distance to microbiome cluster centroid by clinical outcome**. Violin and boxplots showing Bray-Curtis distance to the group centroid for samples classified as regression or progression. Distances were calculated from the beta diversity ordination space to evaluate within-group microbiome dispersion. Statistical significance was assessed using a Mann–Whitney U test.

**Supplementary Figure S3. Microbial diversity and Lactobacillus dominance across CST I subtypes**. (**A**) Alpha diversity (Shannon index) comparing CST I-A and CST I-B samples. (**B**) Relative dominance of *Lactobacillus spp*. in CST I subtypes. Statistical significance was assessed using a Mann–Whitney U test.

**Supplementary Figure S4. Microbial co-occurrence network within CST I-A**. Chord diagram illustrating co-occurrence relationships among bacterial species within CST I-A samples. Each segment represents a microbial taxon, and connecting ribbons indicate positive associations based on co-abundance patterns across samples.

**Supplementary Figure S5**. **Microbial co-occurrence network within CST I-B**. Chord diagram illustrating co-occurrence relationships among dominant bacterial species within CST I-B samples. Each segment represents a microbial taxon, and connecting ribbons indicate positive associations based on co-abundance patterns across samples.

**Supplementary Figure S6. Inferred functional pathway alterations in the cervicovaginal microbiome.** Heatmap of MetaCyc pathway abundances inferred using PICRUSt2 (log-transformed and row-scaled) across samples. Samples are clustered based on functional profiles, with annotation bars indicating clinical outcome (regression vs progression) and community state type (CST).

**Supplementary Figure S7. Inferred functional pathway differences at cohort-wide and CST-specific levels.** (**A**) Inferred functional module differences between CST I-B and CST I-A samples, summarized as differences in median pathway scores. (**B**) Top differentially abundant MetaCyc pathways, as inferred using PICRUSt2, between regression and progression groups in the study cohort, ranked by effect size (log2 fold change). Although several pathways related to carbohydrate metabolism and fermentation exhibit directional shifts, statistical significance was limited across the cohort.

**Supplementary Tables**

**Supplementary Table S1. Baseline high-risk HPV test results according to clinical outcome.**

| **Baseline HPV result** | **Progression (*n* = 45, %)** | **Regression (*n* = 45, %)** |
| --- | --- | --- |
| HPV16 and/or HPV18 | 16 (35.6%) | 12 (26.7%) |
| Other hrHPV, known not to be HPV16/18 | 25 (55.6%) | 30 (66.7%) |
| Generic hrHPV-positive result | 4 (8.9%) | 2 (4.4%) |
| hrHPV-negative | 0 (0.0%) | 1 (2.2%) |

**Supplementary Table S2. Distribution of cervicovaginal community state types according to clinical outcome.**

| **CST** | **Total** | (%) | **Regression** | (%) | **Progression** | (%) |
| --- | --- | --- | --- | --- | --- | --- |
| CST I-A | 17 | 19.50 | 6 | 13.60 | 11 | 25.60 |
| CST I-B | 10 | 11.50 | 8 | 18.20 | 2 | 4.70 |
| CST II | 2 | 2.30 | 1 | 2.30 | 1 | 2.30 |
| CST III-A | 12 | 13.80 | 5 | 11.40 | 7 | 16.30 |
| CST III-B | 13 | 14.90 | 7 | 15.90 | 6 | 14.00 |
| CST IV-A | 6 | 6.90 | 4 | 9.10 | 2 | 4.70 |
| CST IV-B | 22 | 25.30 | 10 | 22.70 | 12 | 27.90 |
| CST IV-C | 1 | 1.10 | 0 | 0.00 | 1 | 2.30 |
| CST V | 4 | 4.60 | 3 | 6.80 | 1 | 2.30 |
| Total | 87 | 100 | 44 | 100 | 43 | 100 |

**Supplementary Table S3. Statistical comparison of inferred MetaCyc pathways displayed in Figure 3 between CST I-A and CST I-B.**

| MetaCyc_ID | Pathway | Median_CST_IA | Median_CST_IB | Log2FC_IB_vs_IA | *P* value | BH_FDR |
| --- | --- | --- | --- | --- | --- | --- |
| LACTOSECAT-PWY | lactose and galactose degradation I | 0 | 0.0072 | 9.09 | 6.38E-05 | 0.00255 |
| PWY-6527 | stachyose degradation | 0.0225 | 0.0113 | -0.999 | 0.000206 | 0.00359 |
| PWY-7221 | guanosine ribonucleotides de novo biosynthesis | 0.0258 | 0.0152 | -0.766 | 0.00027 | 0.00359 |
| PWY-6151 | S-adenosyl-L-methionine salvage I | 0.0225 | 0.0128 | -0.818 | 0.000916 | 0.00916 |
| PWY-5686 | UMP biosynthesis I | 0.0256 | 0.0143 | -0.838 | 0.00387 | 0.031 |
| PWY-6317 | D-galactose degradation I | 0.0258 | 0.0248 | -0.053 | 0.052 | 0.347 |
| PWY0-1586 | peptidoglycan maturation | 0.0547 | 0.0583 | 0.091 | 0.115 | 0.657 |
| PWY-7237 | myo-, chiro- and scyllo-inositol degradation | 0.0216 | 0.0076 | -1.5 | 0.319 | 0.863 |
| TRNA-CHARGING-PWY | tRNA charging | 7.00E-04 | 0.0018 | 1.361 | 0.187 | 0.863 |
| PWY-7790 | UMP biosynthesis II | 0.0256 | 0.0236 | -0.121 | 0.863 | 0.863 |
| PWY-7791 | UMP biosynthesis III | 0.0256 | 0.0236 | -0.121 | 0.863 | 0.863 |
| PWY-2941 | L-lysine biosynthesis II | 0.0224 | 0.0206 | -0.12 | 0.824 | 0.863 |
| PWY-5097 | L-lysine biosynthesis VI | 0.0224 | 0.0206 | -0.119 | 0.824 | 0.863 |
| PWY-2942 | L-lysine biosynthesis III | 0.0224 | 0.0207 | -0.113 | 0.824 | 0.863 |
| DAPLYSINESYN-PWY | L-lysine biosynthesis I | 0.0225 | 0.0208 | -0.109 | 0.824 | 0.863 |
| PWY-7220 | adenosine deoxyribonucleotides de novo biosynthesis II | 0.0225 | 0.0213 | -0.084 | 0.863 | 0.863 |
| PWY-7222 | guanosine deoxyribonucleotides de novo biosynthesis II | 0.0225 | 0.0213 | -0.084 | 0.863 | 0.863 |
| PWY-7229 | adenosine nucleotides de novo biosynthesis I | 0.0026 | 0.0025 | -0.055 | 0.604 | 0.863 |
| GLYCOLYSIS | glycolysis I | 0.0239 | 0.0248 | 0.053 | 0.334 | 0.863 |
| PWY-6471 | peptidoglycan biosynthesis IV | 0.0259 | 0.0267 | 0.043 | 0.359 | 0.863 |
| PWY-6470 | peptidoglycan biosynthesis V | 0.0246 | 0.0254 | 0.042 | 0.359 | 0.863 |
| POLYISOPRENSYN-PWY | polyisoprenoid biosynthesis | 0.0226 | 0.0232 | 0.039 | 0.473 | 0.863 |
| PWY-6385 | peptidoglycan biosynthesis III | 0.0251 | 0.0257 | 0.035 | 0.414 | 0.863 |
| PWY-3841 | folate transformations II | 0.0226 | 0.023 | 0.026 | 0.711 | 0.863 |
| GLUCOSE1PMETAB-PWY | glucose and glucose-1-phosphate degradation | 0.0226 | 0.023 | 0.026 | 0.604 | 0.863 |
| PWY-5667 | CDP-diacylglycerol biosynthesis I | 0.0271 | 0.0276 | 0.025 | 0.711 | 0.863 |
| PWY0-1319 | CDP-diacylglycerol biosynthesis II | 0.0271 | 0.0276 | 0.025 | 0.711 | 0.863 |
| PWY-6859 | all-trans-farnesol biosynthesis | 0.0226 | 0.023 | 0.025 | 0.749 | 0.863 |
| PHOSLIPSYN-PWY | phospholipid biosynthesis I | 0.026 | 0.0265 | 0.025 | 0.711 | 0.863 |
| PWY4FS-7 | phosphatidylglycerol biosynthesis I | 0.0254 | 0.0258 | 0.025 | 0.675 | 0.863 |
| PWY4FS-8 | phosphatidylglycerol biosynthesis II | 0.0254 | 0.0258 | 0.025 | 0.675 | 0.863 |
| PWY-7851 | coenzyme A biosynthesis II | 0.0226 | 0.023 | 0.025 | 0.749 | 0.863 |
| COA-PWY-1 | coenzyme A biosynthesis III | 0.0226 | 0.023 | 0.025 | 0.749 | 0.863 |
| UDPNAGSYN-PWY | UDP-N-acetyl-D-glucosamine biosynthesis I | 0.0226 | 0.023 | 0.025 | 0.749 | 0.863 |
| PWY-6387 | UDP-N-acetylmuramoyl-pentapeptide biosynthesis I | 0.0241 | 0.0245 | 0.025 | 0.749 | 0.863 |
| PEPTIDOGLYCANSYN-PWY | peptidoglycan biosynthesis I | 0.0238 | 0.0242 | 0.025 | 0.749 | 0.863 |
| PWY-7953 | UDP-N-acetylmuramoyl-pentapeptide biosynthesis III | 0.0243 | 0.0247 | 0.024 | 0.749 | 0.863 |
| PWY-6168 | flavin biosynthesis III | 0.0225 | 0.0229 | 0.023 | 0.711 | 0.863 |
| RIBOSYN2-PWY | flavin biosynthesis I | 0.0225 | 0.0229 | 0.023 | 0.711 | 0.863 |
| PWY-7228 | guanosine nucleotides de novo biosynthesis I | 0.0014 | 0.0014 | 0.019 | 0.675 | 0.863 |
